# Metabolic syndrome in hypertensive adults from rural Northeast China: an update

**DOI:** 10.1186/s12889-015-1587-7

**Published:** 2015-03-14

**Authors:** Shasha Yu, Xiaofan Guo, Hongmei Yang, Liqiang Zheng, Yingxian Sun

**Affiliations:** Department of Cardiology, The First Hospital of China Medical University, 155 Nanjing North Street, Heping District, Shenyang, Post code:110001 China; Department of Clinical Epidemiology, Shenjing Hospital of China Medical University, Shenyang, Liaoning China

**Keywords:** Metabolic syndrome, Prevalence, Risk factor, Hypertension

## Abstract

**Background:**

The last study reported the prevalence of Metabolic Syndrome (MetS) in hypertensive residents from rural Northeast China was conducted approximately ten years ago. The purpose of this study was to update the prevalence and epidemiological features of Metabolic syndrome (MetS).

**Methods:**

This survey was conducted from July 2012 to August 2013. In this study, a total of 5866 hypertensive residents from the rural Northeast China were randomly selected and examined. MetS was defined according to the to the International Diabetes Federation (IDF) criteria. Data regarding the demographic and lifestyle characteristics and the blood biochemical indexes of these participants were collected by well-trained personnel.

**Results:**

The overall prevalence of MetS among hypertensive residents was 43.1%. Women had significantly higher incidence of MetS than men (56.4%vs. 29.2%, *P* < 0.001). Overall, 51.7%, 39.6%, 29.3% and 55.9% of the hypertensive adults had abdominal obesity, hypertriglyceridemia, low HDL-C, and increased fasting glucose. Multivariate logistic regression, after adjusting for possible confounders, revealed the following factors that increased the risk of MetS: being female, older age, completion of education through high school, obesity, current smoking. Moderate physical activity, a family income between 5000–20000 CNY per year and higher diet score were correlated with lower rates of MetS.

**Conclusions:**

The prevalence of MetS was dramatically high and exhibited a remarkably increasing trend in hypertensive rural Northeast Chinese. Female had higher incidence of MetS while male had more drastically increasing trend.

## Background

As with other chronic diseases, hypertension is prevalent in China [1]. Data from the China National Nutrition and Health Survey in 2002 showed that the prevalence of hypertension was 25% in Northeast, 27% in North, 17% in Northwest, 12% in Southwest, 19% in East and 17% in Middle South [2]. This study also confirmed that the prevalence of hypertension was higher in urban compared with rural areas in both gender [2]. We performed a multistage cluster random sampling of a group of 29,970 adult residents from 2005 to 2006 and found that the overall prevalence of hypertension was increasing to 36.2% [3]. Previous study investigated that the prevalence of hypertension among middle-aged and old adults in rural Northwest in China from 1982 to 2010 increased from 18.4% to 30.5% [4].

Framingham Heart Study confirmed that hypertensive individuals had more additional risk factors than their counterparts [5]. As we know, MetS is a cluster of cardiovascular risk factors including hypertriglyceridemia, elevated blood pressure, abdominal obesity, increasing fasting glucose and low HDL-C which are all closely linked to insulin resistance [6]. Furthermore, there was study confirmed that the frequency of cardiometabolic risks associating with MetS was greater when hypertension was presented [7].

With the development of economy, high prevalence of MetS becomes an important public health problem in China. The China Health and Nutrition Survey carried in 2009 reported that the prevalence of MetS reached 21.3%, 18.2% and 10.5% based on definitions of revised NCEP ATPIII, IDF and CDS criteria, respectively [8]. Our previous study conducted during 2004–2006 in rural Northeast China found that the prevalence of MetS was higher in the hypertensive patients than in the general population and the overall prevalence of MetS was 36.2%, higher than the general level [9].

In the past ten years, the rural economy in China has achieved a rapid development. However, there was no study focused on the temporal trend of the prevalence and updating the possible risk factors of MetS among hypertensive populations. We hypothesized that huge changes in the prevalence, risk factors of MetS among hypertensive populations might happen in rural Northeast China during this 10 years. Therefore, the objective of this study was to update the prevalence of MetS as well as their risk factors in hypertensive rural Chinese during 2012–2013.

## Methods

### Study population

Liaoning Province is located in Northeast China. From January 2012 to August 2013, a representative sample of participants aged ≥ 35 years was selected to characterize the prevalence, incidence and natural history of cardiovascular risk factors in rural areas of Liaoning Province. The study adopted a multi-stage, stratified, random-cluster sampling scheme. In the first stage, three counties (Dawa, Zhangwu and Liaoyang County) were selected from the eastern, southern and northern regions of Liaoning province. In the second stage, one town was randomly selected from each county (for a total of three towns). In the third stage, 8–10 rural villages from each town were randomly selected (for a total of 26 rural villages). Participants who were pregnant or had malignant tumors or mental disorders were excluded from the study. All the eligible permanent residents aged ≥ 35 years from each village were invited to attend the study (a total of 14,016 participants). Of those, 11,956 participants agreed and completed the study to give a response rate of 85.3%. The study was approved by the Ethics Committee of China Medical University (Shenyang, China). All procedures were performed in accordance with ethical standards. Written consent was obtained from all participants after they had been informed of the objectives, benefits, medical items and confidentiality agreement regarding their personal information. For participants who were illiterate, we obtained written informed consent from their proxies. In this report, we used only the data from participants who completed the study, which provided a final sample size of 5866 (2859 men and 3007 women).

### Data collection and measurements

Data were collected during a single visit to the clinic by cardiologists and trained nurses using a standard questionnaire in a face-to-face interview. Before the survey was performed, we invited all eligible investigators to attend an organized training session. The training included the purpose of this study, how to administer the questionnaire, the standard method of measurement, the importance of standardization and the study procedures. A strict test was administered after this training, and only those who scored perfectly on the test were accepted as investigators in this study. During data collection, our inspectors had further instructions and support.

Data regarding the demographic characteristics, lifestyle risk factors, dietary habits, family income and family history of chronic diseases were obtained during the interview using the standardized questionnaire. The study was guided by a central steering committee with a subcommittee for quality control. Educational level was assessed as completion of primary school or less, middle school or high school and higher. Self-reported sleep duration (including nocturnal and nap duration) was obtained from the questionnaire. The responses were categorized into four groups: ≤7, 7–8, 8–9 and >9 h/d. Family income was classified as ≤5000, 5000–20,000 and >20,000 CNY/year. Self-reported sleep duration (including nocturnal and nap duration) was obtained from the questionnaire. The responses were categorized into four groups: ≤7, 7–8, 8–9, and >9 h/d.

According to American Heart Association protocol, blood pressure (BP) was measured three times at 2-min intervals after at least 5 min of rest using a standardized automatic electronic sphygmomanometer (HEM-907; Omron), which had been validated according to the British Hypertension Society protocol [10]. The participants were advised to avoid caffeinated beverages and exercise for at least 30 min before the measurement. During the measurement, the participants were seated with their arms supported at the level of the heart. The mean of three BP measurements was calculated and used in all analyses.

Weight and height were measured to the nearest 0.1 kg and 0.1 cm, respectively, with the participants wearing light-weight clothing and without shoes. Waist circumference (WC) was measured at the umbilicus using a non-elastic tape (to the nearest 0.1 cm), with the participants standing at the end of normal expiration. Body mass index (BMI) was calculated as the weight in kilograms divided by the square root of the height in meters.

Fasting blood samples were collected in the morning after at least 12 h of fasting. Blood samples were obtained from an antecubital vein into Vacutainer tubes containing ethylenediaminetetraacetic acid (EDTA). Fasting plasma glucose (FPG), total cholesterol (TC), low-density lipoprotein cholesterol (LDL-C), high-density lipoprotein cholesterol (HDL-C), triglycerides (TGs) and other routine blood biochemical indexes were analyzed enzymatically using an autoanalyzer. All laboratory equipment was calibrated, and blinded duplicate samples were used for these analyses.

### Definitions

According to the International Diabetes Federation (IDF) criteria, individuals have the MetS if he/she has central adiposity plus two or more of the following four factors:(1) raised concentration of triglycerides ≥1.7mmol/L or taking abnormal lipid medication; (2)reduced concentration of HDL-C < 1.03mmol/L in male and HDL-cholesterol < 1.29mmol/L in female; (3)elevated blood pressure ≥ 130/85mmHg or taking hypertension medication;(4) increasing fasting glucose: ≥ 5.6mmol/L or taking diabetes medication [11].

Physical activity included occupational and leisure-time physical activity. A detailed description of the methods for assessing physical activity has been presented elsewhere [12]. Occupational and leisure-time physical activity were merged and regrouped into the following three categories: 1) low—subjects who reported light levels of both occupational and leisure-time physical activity, 2) moderate—subjects who reported moderate or high levels of either occupational or leisure-time physical activity and 3) high—subjects who reported a moderate or high level of both occupational and leisure-time physical activity.

Dietary patterns were assessed by having participants recall the foods they had eaten during the previous year. The questionnaire included questions regarding the average consumption of several food items per week. The reported consumption was quantified approximately in terms of grams per week. Vegetable consumption was assessed on the following scale: rarely = 3, <1000 g = 2, 1000–2000 g = 1, ≥2000 g = 0, and meat consumption, including red meat, fish and poultry was assessed on the following scale: rarely = 0, <250 g = 1, 250–500 g = 2 and ≥500 g = 3). A special diet score (vegetable consumption score plus meat consumption score) was calculated for each participant (range 0–6). Higher values of the diet score indicated higher meat consumption, lower vegetable consumption and greater adherence to a Westernized diet, while lower values indicate adherence to the Chinese diet. Similar methods for calculating a diet score can be found in the ATTICA study [13].

### Statistical analysis

Descriptive statistics were calculated for all the variables, including continuous variables (reported as mean values and standard deviations) and categorical variables (reported as numbers and percentages). Differences between healthy and MetS groups were evaluated using Student’s t-test, ANOVA, non-parametric test or the χ2-test as appropriate. Multivariate logistic regression analyses were used to identify independent factors of MetS among hypertensive adults with odds ratios (ORs) and corresponding 95% confidence intervals (CIs) calculated. All the statistical analyses were performed using SPSS version 17.0 software, and P values less than 0.05 were considered to be statistically significant.

## Results

### Basic characteristics of study population

The characteristics of the hypertensive rural adults enrolled in this study, as stratified with MetS or not, are shown in Table 1. Compared with the non-MetS group, individuals with MetS had significantly higher levels of systolic BP, diastolic BP, waist circumference, BMI, fasting plasma glucose, triglycerides, total cholesterol, LDL-C and the proportion of treated individuals, graduating from high school education was also higher in the MetS group. However, prevalence of current drinking and smoking, diet score, and levels of HDL-C and the proportion of moderate physical activity were lower in the individuals with MetS. There was no significant differences of mean age, the proportion of Han, sleep duration and annual income between these two groups.

**Table 1:** **Characteristics of study population with or without MetS in the rural hypertensive population of Liaoning Province, China**

**Variables**	**Total**	**Non-MetS**	**MetS**	***P-*** **value**
	**(n = 5866)**	**(n = 3336)**	**(n = 2530)**	
**Age (year)**	57.29 ± 10.24	57.48 ± 10.50	57.04 ± 9.89	0.109
**Ethnicity**				0.128
Han	5555(94.7)	3149(94.4)	2406(95.1)	
Others^a^	311(5.3)	187(5.6)	124(4.9)	
**Educational status**				0.001
Primary school or below	3276(55.8)	1803(54.0)	1473(58.2)	
Middle school	2075(35.4)	1248(37.4)	827(32.7)	
High school or above	515(8.8)	285(8.5)	230(9.1)	
**Physical activity**				<0.001
Light	1993(34.0)	1025(30.7)	968(48.6)	
Moderate	3531(60.2)	2134(64.0)	1397(55.2)	
Severe	342(5.8)	177(5.3)	165(6.5)	
**Annual income (CNY/year)**				0.081
≤5000	897(15.3)	520(15.6)	377(42.0)	
5000-20000	3282(55.9)	1895(56.8)	1387(54.8)	
>20000	1687(28.8)	921(27.6)	766(30.3)	
**Smoking status (%)**				<0.001
Never	3492(59.5)	1786(53.5)	1706(48.9)	
Former	318(5.4)	195(5.8)	123(4.9)	
Current	2056(35.0)	1355(65.9)	701(34.1)	
**Drinking status (%)**				<0.001
Never	4237(72.2)	2197(65.9)	2040(80.6)	
Former	196(3.3)	125(3.7)	71(2.8)	
Current	1433(24.4)	1014(30.4)	419(16.6)	
**Diet score**	2.30 ± 1.13	2.37 ± 1.12	2.20 ± 1.14	<0.001
**Sleep duration (h/d)**	7.23 ± 1.76	7.26 ± 1.75	7.20 ± 1.78	0.276
**SBP (mmHg)**	158.91 ± 19.66	157.61 ± 18.87	160.62 ± 20.53	<0.001
**DBP (mmHg)**	88.85 ± 11.17	88.14 ± 10.89	89.79 ± 11.47	<0.001
**BMI (kg/m2)**	25.56 ± 3.66	23.90 ± 3.05	27.76 ± 3.21	<0.001
**WC (cm)**	84.89 ± 9.74	79.82 ± 8.00	91.57 ± 7.54	<0.001
**TC (mmol/L)**	5.42 ± 1.11	5.30 ± 1.04	5.57 ± 1.19	<0.001
**TG (mmol/L)**	1.83 ± 1.66	1.44 ± 1.18	2.35 ± 2.02	<0.001
**LDL-C (mmol/L)**	3.08 ± 0.86	2.97 ± 0.82	3.22 ± 0.89	<0.001
**HDL-C (mmol/L)**	1.42 ± 0.40	1.53 ± 0.43	1.28 ± 0.31	<0.001
**FPG (mmol/L)**	6.16 ± 1.89	5.83 ± 1.57	6.60 ± 2.17	<0.001
**Antihypertensive medication**				
Yes	1733(29.5)	746(22.3)	987(39.0)	<0.001

Data are expressed as the mean ± SD or as n (%). Abbreviations: BMI, body mass index; WC, waist circumference; CNY, China Yuan (1CNY = 0.161 USD); SBP, systolic blood pressure; DBP, diastolic blood pressure; TC, total cholesterol; TG, triglyceride; LDL-C, low-density lipoprotein cholesterol; HDL-C, high-density lipoprotein cholesterol; FPG, fasting plasma glucose.^a^ Including some ethnic minorities in China, such as Mongol and Manchu.

### Prevalence of MetS and its components

**Figure 1:**
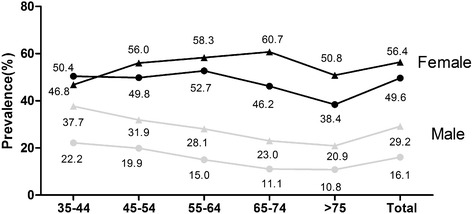
**The prevalence of MetS in different age group stratified by gender.** The triangle blot means 2012–2013 years while the circular blot means 2004–2006 years.

As shown in Figure 1, approximately 43.1% of the hypertensive adults had MetS (Male: 29.2% ,Female: 56.4%). The highest prevalence of MetS was in the age of 35–44 years in male and in the age of 65–74 years in female. Increased trend of the prevalence of MetS in rural hypertensive Northeastern Chinese for our sample using the same criteria [9] during 2004–2013 years was observed in Figure 1.

**Figure 2:**
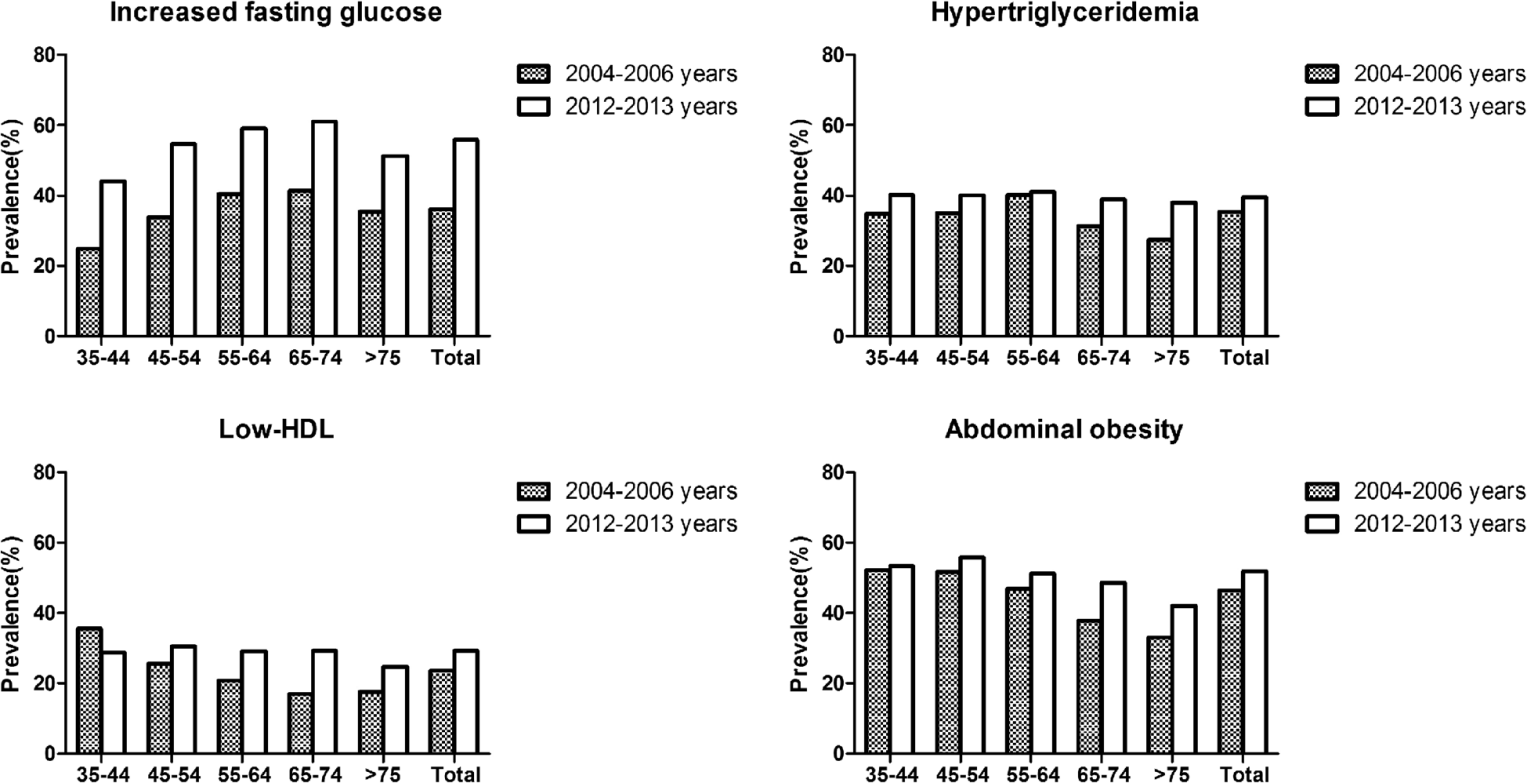
**The increasing trend of the prevalence of different metabolic components from 2004 to 2013 stratified by age.**

Figure 2 presented the prevalence of each component of the MetS. In this sample, the prevalence of abdominal obesity, hypertriglyceridemia, low HDL-C, and increasing fasting plasma glucose was 51.9%, 39.6%, 29.3% and 55.9%. Furthermore, the most common metabolic disorder in our study was increasing fasting plasma glucose and followed by abdominal obesity which was closed to our previous study [9].

### Factors associated with MetS and metabolic components

Table 2 shown the ORs for the influencing factors associated with MetS and metabolic components among the hypertensive adults. Individuals who were women [OR(95%CI):4.78(4.0,5.69)], 45 years or older [OR(95%CI):1.27(1.02,1.58) for 45–54 years; OR(95%CI):1.66(1.33,2.08) for 55-64years; OR(95%CI):1.89(1.45,2.47) for 65–74 years, OR(95%CI):1.99(1.36,2.95) for >75years], graduated from high school or above [OR(95%CI):1.30(1.01,1.66)], obesity [OR(95%CI):1.60(1.56,1.65)] and current smoking [OR(95%CI):1.36(1.16,1.59)] were more likely to have MetS. And moderate physical activity [OR(95%CI):0.85(0.73,0.98)], annual income between 5000–20000 CNY/year [OR(95%CI):0.79(0.66,0.97)], higher diet score [OR(95%CI):0.92(0.86,0.97)] were found to be inversely associated with the probability of MetS.

**Table 2:** **Multiple logistic regression analysis of MetS and the associated factors based on IDF criteria among rural hypertensive population in Liaoning Province, China**

**Variables**	**OR**	**95%CI**	***P*** **-value**
**Female**	4.78	4.0,5.69	<0.001
**Age group**			
35-44	1.00(reference)		
45-54	1.27	1.02,1.58	0.036
55-64	1.66	1.33,2.08	<0.001
65-74	1.89	1.45,2.47	<0.001
>75	1.99	1.36,2.95	<0.001
**Ethnicity**			
Han	1.00(reference)		
Others^a^	0.66	0.48,0.89	0.006
**Educational status**			
Primary school or below	1.00(reference)		
Middle school	1.03	0.88,1.20	0.745
High school or above	1.30	1.01,1.66	0.040
**Physical activity**			
Light	1.000(reference)		
Moderate	0.85	0.73,0.98	0.028
Severe	1.17	0.87,1.56	0.301
**Annual income (CNY/year)**			
≤ 5000	1.000(reference)		
5000-20000	0.79	0.66,0.97	0.026
>20000	0.89	0.72,1.12	0.340
**Sleep duration (h/d)**			
≤7	1.00(reference)		
7-8	0.97	0.83,1.14	0.743
8-9	0.91	0.75,1.11	0.361
>9	1.04	0.81,1.33	0.773
**Diet score (per 1 unit)**	0.92	0.86,0.97	0.005
**BMI (per 1 unit)**	1.60	1.56,1.65	<0.001
**Smoking status (%)**			
Never	1.00(reference)		
Former	1.23	0.91,1.67	0.186
Current	1.36	1.16,1.59	<0.001
**Drinking status (%)**			
Never	1.00(reference)		
Former	1.06	0.72,1.56	0.765
Current	0.99	0.82,1.21	0.983

Abbreviations: OR, odds ratio; 95% CI, 95% confidence interval; CNY, China Yuan (1CNY = 0.161 USD).^a^ Including some ethnic minorities in China, such as Mongol and Manchu.

## Discussion

The present study investigated that 43.1% of the hypertensive adults in rural Northeast China had MetS. Female had significantly higher incidence of MetS than male (56.4% for female and 29.2% for male, *P* < 0.001). An increasing prevalence of MetS was seen in both gender, especially in male. Increasing fasting glucose and abdominal obesity was the most common metabolic disorder in both gender. Age, gender, education, obesity, annual income, physical activity, current smoking and higher diet score was significantly associated with MetS.

Similar to those previous studies, our study investigated that MetS in hypertensive individuals was more prevalent than in the general population (43.1% vs. 30.9%) using the IDF criteria [7]. The Korean National Health and Nutrition Examination Survey reported that the prevalence of MetS was doubled in hypertensive population in compared to normotensive population as well [14]. Other studies also demonstrated that MetS was prevalent in hypertensive adults, like Nigeria (42.5%), India (63.5%), Brazil (82.4%) and Iran (51.6%) [14-17]. Insulin resistance plays a key role in this situation [18]. The sex-specific prevalence in our study was 29.2% and 56.4% among males and females respectively. Similarly, hypertensive women in Iran (65.3% vs. 33.5%) and India (47.6% vs. 15.1%) had higher possibility to have MetS than men as well [16,19]. On one hand, the possible reason for the high prevalence of MetS in hypertensive female might be the menopause [20]. As seen in our study the highest incidence of MetS in hypertensive female was older than 55 years old. Previous study demonstrated that the incidence of MetS increased with menopause [21]. On the other hand, traditional Chinese cultural influences discouraged women from outdoor activities and very few women took physical activities. In our study, female had significantly higher incidence of low physical activity than male (40.5% vs. 27.1%, *P* < 0.001).

Hiram Beltrán-Sánchez and colleagues reported that the prevalence of MetS in the U.S adult population decreased from 25.5% to 22.9% from 1999 to 2010. Moreover, hypertriglyceridemia (33.5% to 24.5%) and elevated blood pressure (32.3% to 24.0%) prevalence also decreased during the past ten years [22]. At the same time, study carried in Korea confirmed that the prevalence of hypertension as well as MetS among the general population decreased slightly during the past ten years. However, MetS prevalence among the hypertensive population continued to rise [23]. Our study also reached the same conclusion that MetS in hypertensive adults increased from 2004 to 2012 which was more obvious among older patients in men than in women. One interesting finding was that during the past years, prevalence of MetS in hypertensive increased more drastically in male (from 16.1% to 29.2%) than in female (from 49.6% to 56.4%) using the same criterion in the rural Northeast China. And we still need further study to explain this finding. The high prevalence of MetS among hypertensive individuals required the need for metabolic screening in all newly diagnosed hypertensive individuals, especially in hypertensive males in rural areas.

The most common phenotype of MetS with the component of hypertension was the coexistence of increasing fasting glucose and abdominal obesity in rural Northeast Chinese. This was quite different from other countries like Nigeria, India and America. In Nigerian and Indian, visceral obesity and low HDL-C were the other common cardiovascular risk factors among hypertensive subjects [14,15]. While in America, besides elevated blood pressure, abdominal obesity and hypertriglyceridemia was the most common metabolic disorder. The possible reason accounted for this situation might be the high prevalence of diabetes and prediabetes in China. A national study from 2007 to 2008 discovered that 92.4 million Chinese adults with diabetes (50.2 million men and 42.2 million women) and 148.2 million Chinese adults with prediabetes (76.1 million men and 72.1 million women) [24]. Having such a large population of diabetes and prediabetes given above some experts draw a conclusion that China may bear a higher increasing fasting glucose burden than any other country [25].

Our study investigated that minority, mostly Mongolian and Huis, had less possibility to have MetS than Han. This conclusion was inline with our previous study that Mongolian had relatively lower triglyceride level than Han [26]. Due to the small amount of minority in the present research, further study was needed to confirmed this relation. While most studies found an inverse association between education status and MetS, on the contrary, higher prevalence of MetS was positively related with higher education in our study after adjusted for age which somehow the same as Jamaican adults [27]. We also demonstrated that individuals with moderate physical activity had less possibility to had MetS and the possible reason might be moderate physical activity help improve individual metabolic parameters and lose ones weight [28]. However, vigorous physical activity was not related to MetS in our study because only 5.8% of the participants took part in vigorous physical activity in rural Northeast China. Annual income was associated with MetS in our study. But the relationship between income and MetS or other cardiovascular risks were still controversial in developing and developed countries [29]. In compared with never smoking, current smoking increased the risk of MetS which had been well proven by previous study [30].

### Limitations

First, our study was a cross-sectional study suggesting that it cannot provide sufficient evidence of causality. Longitudinal studies were required for the further investigation. Second, miss classification might exist because the prevalence of MetS was based on a single assessment of blood. Third, although the researchers had been trained according to a standardized protocol of measurements, there might be incorrect values for the anthropometric indexes due to the single visit. Other limitations include recall bias and the small sample sizes available in some subgroup analyses that reduced the statistical power to detect significant associations. Fourth, in our study, we enrolled only the hypertensive patients which was not comprehensive.

## Conclusion

The prevalence of MetS in hypertensive rural Northeast China had increase dramatically during the past ten years. Over 50% of hypertensive females had MetS in rural Northeast China. Furthermore, the obviously increasing trend of MetS during the past ten years in hypertensive males deserved more attention. Metabolic screening was in-need in all newly diagnosed hypertensive individuals, especially in hypertensive male.

## Abbreviations

MetS: Metabolic syndrome
CNY: China Yuan
WC: Waist circumference
BMI: Body mass index
FPG: Fasting plasma glucose
TC: Total cholesterol
LDL-C: Low-density lipoprotein cholesterol
HDL-C: High-density lipoprotein cholesterol
TG: Triglyceride
SBP: Systolic blood pressure
DBP: Diastolic blood pressure

## References

[1] Gao Y, Chen G, Tian H, Lin L, Lu J, Weng J, Jia W, Ji L, Xiao J, Zhou Z, Ran X, Ren Y, Chen T, Yang W (2013). Prevalence of hypertension in china: a cross-sectional study. PLoS One.

[2] Wu Y, Huxley R, Li L, Anna V, Xie G, Yao C (2008). Prevalence, awareness, treatment, and control of hypertension in China: data from the China National Nutrition and Health Survey 2002. Circulation.

[3] Dong G, Sun Z, Zheng L, Li J, Zhang X, Zhang X (2007). Prevalence, awareness, treatment, and control of hypertension in rural adults from Liaoning Province, northeast China. Hypertens Res.

[4] Zhao Y, Yan H, Marshall RJ, Dang S, Yang R, Li Q, Qin X (2013). Trends in population blood pressure and prevalence, awareness, treatment, and control of hypertension among middle-aged and older adults in a rural area of Northwest China from 1982 to 2010. PLoS One.

[5] Weycker D, Nichols GA, O’Keeffe-Rosetti M, Edelsberg J, Khan ZM, Kaura S, Oster G (2007). Risk-factor clustering and cardiovascular disease risk in hypertensive patients. Am J Hypertens.

[6] Haffner S, Taegtmeyer H (2003). Epidemic obesity and the metabolic syndrome. Circulation.

[7] Marchi-Alves LM, Rigotti AR, Nogueira MS, Cesarino CB, de Godoy S (2012). Metabolic syndrome components in arterial hypertension. Rev Esc Enferm USP.

[8] Xi B, He D, Hu Y, Zhou D (2013). Prevalence of metabolic syndrome and its influencing factors among the Chinese adults: the China Health and Nutrition Survey in 2009. Prev Med.

[9] Zhang X, Sun Z, Zhang D, Zhu R, Zheng L, Liu S (2007). Prevalence of metabolic syndrome among rural population with hypertension in Fuxin of Liaoning Province. Chin J Endocrinol Metab.

[10] O’Brien E, Petrie J, Littler W, de Swiet M, Padfield PL, O’Malley K (1990). The British Hypertension Society protocol for the evaluation of automated and semi-automated blood pressure measuring devices with special reference to ambulatory systems. J Hypertens.

[11] Alberti KG, Zimmet P, Shaw J, IDF Epidemiology Task Force Consensus Group (2005). The metabolic syndrome–a new worldwide definition. Lancet.

[12] Hu G, Tuomilehto J, Silventoinen K, Barengo N, Jousilahti P (2004). Joint effects of physical activity, body mass index, waist circumference and waist-to-hip ratio with the risk of cardiovascular disease among middle-aged Finnish men and women. Eur Heart J.

[13] Panagiotakos DB, Pitsavos C, Chrysohoou C, Risvas G, Kontogianni MD, Zampelas A (2004). Epidemiology of overweight and obesity in a Greek adult population: the ATTICA Study. Obes Res.

[14] Akintunde AA, Ayodele OE, Akinwusi PO, Opadijo GO (2011). Metabolic syndrome: comparison of occurrence using three definitions in hypertensive patients. Clin Med Res.

[15] Thakur S, Raina S, Thakur S, Negi PC, Verma BS (2013). Prevalence of metabolic syndrome among newly diagnosed hypertensive patients in the hills of Himachal Pradesh, India. Indian J Endocrinol Metab.

[16] Kelishadi R, Derakhshan R, Sabet B, Sarraf-Zadegan N, Kahbazi M, Sadri GH (2005). The metabolic syndrome in hypertensive and normotensive subjects: the Isfahan Healthy Heart Programme. Ann Acad Med Singapore.

[17] Bulhões K, Araújo L (2007). Metabolic syndrome in hypertensive patients: correlation between anthropometric data and laboratory findings. Diabetes Care.

[18] Guo S (2014). Insulin signaling, resistance, and the metabolic syndrome: insights from mouse models into disease mechanisms. J Endocrinol.

[19] Osuji CU, Omejua EG (2012). Prevalence and characteristics of the metabolic syndrome among newly diagnosed hypertensive patients. Indian J Endocrinol Metab.

[20] Ross LA, Polotsky AJ (2012). Metabolic correlates of menopause: an update. Curr Opin Obstet Gynecol.

[21] Jouyandeh Z, Nayebzadeh F, Qorbani M, Asadi M (2013). Metabolic syndrome and menopause. J Diabetes Metab Disord.

[22] Beltrán-Sánchez H, Harhay MO, Harhay MM, McElligott S (2013). Prevalence and trends of metabolic syndrome in the adult U.S. population, 1999–2010. J Am Coll Cardiol.

[23] Lee SR, Cha MJ, Kang DY, Oh KC, Shin DH, Lee HY (2013). Increased prevalence of metabolic syndrome among hypertensive population: ten years’ trend of the Korean National Health and Nutrition Examination Survey. Int J Cardiol.

[24] Yang W, Lu J, Weng J, Jia W, Ji L, Xiao J (2010). Prevalence of diabetes among men and women in China. N Engl J Med.

[25] Wild S, Roglic G, Green A, Sicree R, King H (2004). Global prevalence of diabetes: estimates for the year 2000 and projections for 2030. Diabetes Care.

[26] Zhang X, Sun Z, Zhang X, Zheng L, Li J, Liu S (2007). Prevalence of metabolic syndrome in Han and Mongolian rural population with hypertension. J Int Med Res.

[27] Gupta R, Kaul V, Agrawal A, Guptha S, Gupta VP (2010). Cardiovascular risk according to educational status in India. Prev Med.

[28] Bergstrom G, Behre C, Schmidt C (2012). Increased leisure-time physical activity is associated with lower prevalence of the metabolic syndrome in 64-year old women with impaired glucose tolerance. Angiology.

[29] Shariff ZM, Sulaiman N, Jalil RA, Yen WC, Yaw YH, Taib MN (2014). Food insecurity and the metabolic syndrome among women from low income communities in Malaysia. Asia Pac J Clin Nutr.

[30] Zhang L, Guo Z, Wu M, Hu X, Xu Y, Zhou Z (2013). Interaction of smoking and metabolic syndrome on cardiovascular risk in a Chinese cohort. Int J Cardiol.

